# Items, News and Gossip

**Published:** 1872-02

**Authors:** 


### Items, News and Gossip.

This item is going the rounds of the secular press: The following is an incident of the Prince of Wales's illness: " The Prince having been unable to sleep during nearly two days and nights, a hop pillow was placed under his head, and after some hours he sank into a fitful sleep. The hop pillow has been continued, and there is reason to believe its soporific effect has been felt in the repose which has at last brought with it the hope of recovery. During the time when death seemed imminent, a butcher stood in an adjoining room with a lamb, ready at a moment's notice to flay it, that the warm and reeking skin might swathe the Prince's extremities, should they have shown signs of death-coldness." The hop pillow is well enough, but how about the lamb? Were the medical high priests likewise to inspect its entrails? The old feudal law was, that the lord on returning cold from the chase or journeying, might not kill more than two of his serfs in order to warm his feet in their bowels. Surely some loyal Englishman ought to have had "bowels of compassion" and devotion enough to have offered his own integument and abdomen in place of the lamb's, when royalty became "reduced to extremities." This came very near being a lam(b)entable case. —— Subcutaneous injection of morphia is highly commended by Dr. Patterson, of Constantinople, in cholera. He does not think it a specific, but that its beneficial influence is more decided than any other treatment he has seen. —— The hydrocarbon, Xylol, it is said, has been used in Berlin in the treatment of small-pox with good results. Theoretically it is supposed to be taken up by the blood, and thus acts as a disinfectant. It is best given in capsules containing from three to twelve drops each. One of these to be exhibited every one, two or three hours. Xylol is prepared from coal naphtha. It is scarcely probable that it is materially superior to creosote, cajeput, medicinal naphtha, etc., etc. —— Owing to an alleged interference of the publishers with the text of the *National Medical Journal*, the editors have withdrawn from their position, and its publication is temporarily suspended, and the editors, it is understood, are about to start a new journal. —— Among the recent deaths abroad we note the demise of the celebrated ophthalmologist, Prof. JAEGER, at the ripe age of eighty-eight. He continued to perform the most delicate operations until a very advanced age. —— Among the deaths in this country, those of Prof. Charles A. Lee, at Peekskill, N. Y., and Henry D. Bulkley, M.D., of New York City, will be most widely regretted, as their memories will everywhere be cherished. —— In a discussion in the Atlanta Medical Society, it was argued that by appropriate hygienic management, parturition should always be painless, or even, from analogy to the expulsive efforts of the bladder and bowels, positively pleasurable. The morbid irritability and rigidity, Dr. Wilson thought, are superinduced by bad habits of living. A simple mode of living is certainly best in all cases. —— The aboriginal practice of pressure, externally, upon the uterus, in order to facilitate the expulsion of its contents, is being strongly urged by several recent writers. —— It is stated that in the Prussian army vaccination is preferably performed with lymph taken from vesicles of *re*-vaccination. This is believed more powerfully prophylactic in result than when the lymph is taken from children vaccinated for the first time. The writer's own experience is utterly opposed to this view of the case. —— Prof. Huxley is visiting Egypt for his health, and possibly for the inspection of the native mummy. —— During the seige of Paris, the veterinary authorities condemned white horses as unsuitable for eating—the color being regarded as evidencing a lymphatic temperament. And now how about cattle, sheep, hogs, fowls, etc.? They possibly "*do* these things better in France." —— It is estimated that there are 700,000 deaf and dumb people in the world. The number of *males* thus affected considerably preponderates. —— Castor oil is said to be made palatable by combining it with an equal amount of glycerin in which a few drops of oil of cinnamon have been rubbed up. —— A Boston writer commends for the abortive treatment of felons, first soaking the finger in a strong solution of carbolic acid, and then applying several coatings of collodion over the painful part. —— Chloral is being recommended in dram doses in *Cholera*, and in twenty-grain doses to check the profuse sweating accompanying marsh fever. "Next!" —— One part of carbolic acid to eight of sweet oil, is advised as a local application to prevent pitting in small pox. —— Dr. John Shrady (*Med. Record*) praises Ipecacuanha in severe cases of epistaxis, especially when connected with chronic alcoholism. He uses the Vin. Ip. in teaspoonful doses until free emesis is produced. —— Bromide of Potassium, continued in dram doses three times daily for months, is very apt to produce a series of boils, and Dr. Levy writes from Berlin, that these may be prevented by combining with it some preparation of cinchona, —— Prof. Wm. A. Hammond commends the treatment of bedsores by galvanism. A thin silver plate is put over the sore, and this is connected, by a fine silver or copper wire six or eight inches in length, with a zinc plate of the same size on some part of the skin above, a piece of chamois leather soaked in vinegar intervening. Large sores are thus healed in a very short period—the process of granulation observably commencing in a few hours, large sores commencing to cicatrize within forty-eight hours. —— Dr. Turner

(*The Clinic*) urges the use of solid rather than liquid food in typhoid fever. "Give the patient at his usual meal times—thus observing the habits of the digestive system—a regular diet of roasted potatoes, buttered toast, broiled beef steak, game or any other digestible meat, well served and properly seasoned. Give little or no fluids while eating. Encourage salivation by thorough mastication." Though the patient loathes food at the time, persistent efforts should be made to induce him to swallow a few mouthfuls. Usually, in a few days he will begin to relish his nutriment. And in these propositions, also, there are several grains of truth which we sincerely commend to our reader's reflections.

